# Inter- and Intraspecific Variability in Non-Starch Polysaccharide Composition of *Satureja* Species from Tunisia: Implications for Functional Food Development

**DOI:** 10.3390/foods15030525

**Published:** 2026-02-03

**Authors:** Anhar Raadani, Amel Hamdi, Islem Yangui, Ana Jiménez-Araujo, Rocío Rodríguez-Arcos, Imen Ben Elhadj Ali, Rafael Guillén-Bejarano, Chokri Messaoud

**Affiliations:** 1 Laboratory of Nanobiotechnology and Valorisation of Medicinal Phytoresources, National Institute of Applied Sciences and Technology, Department of Biology, Carthage University, B.P. 676, Tunis Cedex 1080, Tunisia; raadanianharbio@gmail.com (A.R.); imenbenelhadjali@yahoo.fr (I.B.E.A.); chok.messaoud@yahoo.fr (C.M.); 2 Phytochemicals and Food Quality Group, Instituto de la Grasa, Consejo Superior de Investigaciones Científicas (CSIC), Pablo de Olavide Universitary Campus, Building 46, Carretera de Utrera Km 1, 41013 Seville, Spain; araujo@ig.csic.es (A.J.-A.); rrodri@ig.csic.es (R.R.-A.); 3 Department of Plant Protection and Biological Sciences, Higher Institute of Agronomy of Chott-Meriem, University of Sousse, Sousse 4042, Tunisia; yanguiislam@gmail.com; 4 Laboratory of Management and Valorization of Forest Resources, National Research Institute for Rural Engineering, Water and Forests, University of Carthage, Ariana 2080, Tunisia; 5 Higher Institute of Biotechnology of Beja, Jendouba University, Beja 9000, Tunisia

**Keywords:** non-starch polysaccharides, soluble fiber, dietary fiber, *Satureja* species, Lamiaceae, chemotaxonomy, principal component analysis, functional foods, Tunisia

## Abstract

Non-starch polysaccharides, the primary structural component of dietary fiber, play critical roles in metabolic and digestive health through multiple physiological mechanisms, yet their composition in Mediterranean aromatic plants remains poorly characterized, limiting the development of novel functional food ingredients. This study provides the first comprehensive NSP profiling of 22 populations across three Tunisian *Satureja* species (*S. nervosa*, *S. graeca*, and endemic *S. barceloi*), using enzymatic analysis, gas chromatography, and multivariate statistics. Total non-starch polysaccharides reached exceptional levels (21.5 ± 3.0 g/100 g dry weight (DW)), with several populations exhibiting unprecedented soluble fiber proportions exceeding 50%, including population SG4 achieving 79.7%. Monosaccharide analysis revealed uronic acid dominance (42.9–52.5% of total NSP), indicating pectin-rich cell walls with distinct functional properties. Principal component analysis (explaining 61.5–84.9% of variance) demonstrated that populations cluster by fiber chemotype rather than taxonomic classification. Hierarchical and K-means clustering identified three distinct clusters in the soluble and total fiber fractions, with uronic acid-dominated populations (SG4, SB, SG18, SN8) and arabinose–xylose enriched populations (SN13, SN12, SN22, SG21) as extreme chemotypes. Intraspecific variation (coefficient of variation, CV: 14.0–50.0%) substantially exceeded interspecific differences. These findings establish Tunisian *Satureja* as an exceptional functional fiber source and demonstrate that population-level chemical screening outperforms taxonomic classification for developing nutraceuticals targeting cholesterol reduction, glycemic control, and gut microbiome modulation.

## 1. Introduction

The human diet plays a key role in health preservation today, given its strong link to chronic diseases such as obesity, type 2 diabetes, and cardiovascular problems. Consequently, prioritizing the consumption of healthy, natural food sources is essential for maintaining well-being. Among these, sources rich in dietary fiber are highly recommended due to their association with a reduced risk of metabolic and gastrointestinal diseases, including cardiovascular diseases, diabetes, obesity, and colorectal cancer [1]. Dietary fibers, consisting of non-starch polysaccharides (NSPs) and lignin, are plant-based components that are not readily digested by human digestive enzymes and play a crucial role in digestive and metabolic health. NSPs are composed of structural units, including various simple sugars (e.g., arabinose, xylose, glucose, galactose, and mannose) and uronic acids (e.g., glucuronic and galacturonic acids). Cellulose is a predominant NSP, widely found in the cell walls of monocotyledonous and dicotyledonous plants [2]. They have distinct physiological properties depending on whether they are soluble or insoluble in water, as their solubility determines their behavior in the gastrointestinal tract [3,4]. Additionally, the influence of NSPs in the gastrointestinal tract is related to several factors, including their quantity, physicochemical composition, and structural properties.

Insoluble NSPs, such as cellulose and hemicellulose, do not dissolve in water but absorb moisture, aiding in stool formation and promoting regular bowel movements. These NSPs are minimally fermented by gut bacteria and are predominantly found in whole grains, vegetables, and fruits with edible skins. They help prevent constipation and maintain colon health by increasing stool bulk and accelerating intestinal transit [5]. Conversely, soluble NSPs, including pectin and gums, dissolve in water to form a gel-like substance. They are partially or fully fermented by gut bacteria, producing short-chain fatty acids beneficial for gut health. These fiberrs slow digestion, regulate blood sugar and cholesterol levels, and support a healthy gut microbiota. Sources of soluble fibre include oats, legumes, fruits, and certain vegetables [6].

Plant NSPs demonstrate considerable potential for application in drug delivery systems and as food additives. Additionally, they hold a generally recognized as safe (GRAS) status for human consumption [7]. Therefore, they are gaining increased attention from the industry for use in pharmaceuticals, biomaterials, food additives, and nutrition [8], with recent advances in extraction and characterization methods enabling better classification and utilization of fibrous ingredients from diverse plant sources [9]. In this context, many medicinal plants have been studied for their NSP contents.

Medicinal plants, especially those belonging to the Lamiaceae family, are recognized as a rich source of non-starch polysaccharides (NSPs), which are widely known for their significant health benefits. These complex, non-digestible carbohydrates demonstrate a range of biological activities, including antioxidant, immunomodulatory, and anti-inflammatory properties. Among the most studied species is *Origanum dictamnus*, an endemic plant of Crete [10,11,12]. Beyond *O. dictamnus,* numerous other Lamiaceae species have been identified as containing bioactive polysaccharides with substantial therapeutic activities, such as *Salvia officinalis* (sage) [13], *Thymus vulgaris* (thyme) [14], *Ocimum basilicum* (basil) [15], and *Mentha piperita* (peppermint) [16]. In this context, a recent patent landscape study highlighted the growing interest in these compounds for the development of novel functional foods, positioning Lamiaceae species as promising sources of dietary fiber and NSPs [11]. Furthermore, a comprehensive review on medicinal plant polysaccharides bridges traditional ethnobotanical knowledge with modern scientific research, emphasizing the central role of NSPs in the pharmacological effects observed in several Lamiaceae species [12].

Within the Lamiaceae family, the fiber found in *Satureja frivaldszkyana* is primarily composed of insoluble fibers. Although the dietary fiber content of *S. frivaldszkyana* is not directly reported, its carbohydrate composition suggests a significant contribution to dietary fiber.

Additionally, *Satureja hortensis* is recognized for its high content of dietary fiber. The fresh leaves of this plant contain approximately 4.45% sugars, while the dried leaves have a dietary fiber content of 8.60% on a dry weight basis. This high fiber content, combined with its richness in vitamins and minerals, highlights its potential as a functional food ingredient. Furthermore, this fiber content is comparable to that of certain cereals and legumes, making it an interesting source of dietary fiber [17].

The plant cell wall is primarily composed of polysaccharides, including cellulose, hemicelluloses, and pectins. It also contains phenolic compounds, which are typically bound to polysaccharides and lignins through ester and ether linkages. The relationship between the chemical structure of these components and their biological functions has been widely studied [18,19,20]. However, the structure of the cell walls in the aerial parts of *Satureja* species remains poorly documented. To the best of our knowledge, this is the first report of the NSP composition of *Satureja* species.

The objectives of this study were to evaluate and compare dietary-fiber composition across 22 populations of three *Satureja* species (*S. barceloi*, *S. graeca*, and *S. nervosa*) and to identify patterns of chemical diversity. Specifically, this involved the determination of (i) total non-starch polysaccharides (TNSPs), insoluble non-starch polysaccharides (INSPs), soluble non-starch polysaccharides (SNSPs), and starch content; (ii) the monosaccharide composition of dietary fiber fractions; and (iii) chemical relationships among populations using multivariate statistical analyses, including principal component analysis, hierarchical clustering, and K-means clustering. These analyses aimed to identify inter- and intraspecific variability in dietary fiber profiles, establish chemotype-based classification systems independent of taxonomic boundaries, and evaluate the potential of *Satureja* populations as sources of functional dietary fibers for nutraceutical applications. Understanding this chemical diversity can provide insights into the nutritional and functional potential of these medicinal plants and guide targeted selection strategies for developing specialized functional food ingredients.

## 2. Materials and Methods

### 2.1. Plant Material and Sampling

Twenty-two populations of three *Satureja* species were collected from different bioclimatic zones across Tunisia: *S. barceloi* (one population), *S. nervosa* (fifteen populations), and *S. graeca* (six populations). The limited sampling of *S. barceloi* (n = 1) reflects the restricted distribution and rarity of this Tunisian endemic species. Consequently, data from *S. barceloi* are reported descriptively without statistical inference, and taxonomic interpretations involving this species should be considered preliminary pending expanded population sampling.

The collection sites spanned from lower-humid to mid-semi-arid zones, representing the natural distribution range of these species in Tunisia (Table S1). Voucher specimens were deposited at the herbarium of the National Institute of Applied Sciences and Technology, Tunis. Aerial parts of plants were harvested during the flowering stage, air-dried at room temperature (22 ± 2 °C) for 7 days, and ground to a fine powder (≤0.5 mm) using a laboratory mill. Ground samples were stored in sealed containers at 4 °C until analysis.

### 2.2. Cell Wall Material Isolation

CWM was extracted by adapting the modified methodology of Selvendran [21]. Briefly, 0.5 g of dried plant material was mixed with 15 mL of aqueous ethanol (80:20, *v*/*v*) and homogenized using an Ultra-Turrax homogeniser (T25, IKA Labortechnik, Staufen, Germany) at maximum speed for 2 min. The mixture was filtered through Whatman No. 1 filter paper, and the residue was subjected to a second extraction under identical conditions. The final residue was washed with absolute ethanol, air-dried overnight, named as alcohol-insoluble residue (AIR), and stored in sealed vials at room temperature until analysis.

### 2.3. Non-Starch Polysaccharide Determination

#### 2.3.1. Sample Preparation and Fractionation Strategy

**Figure 1:**
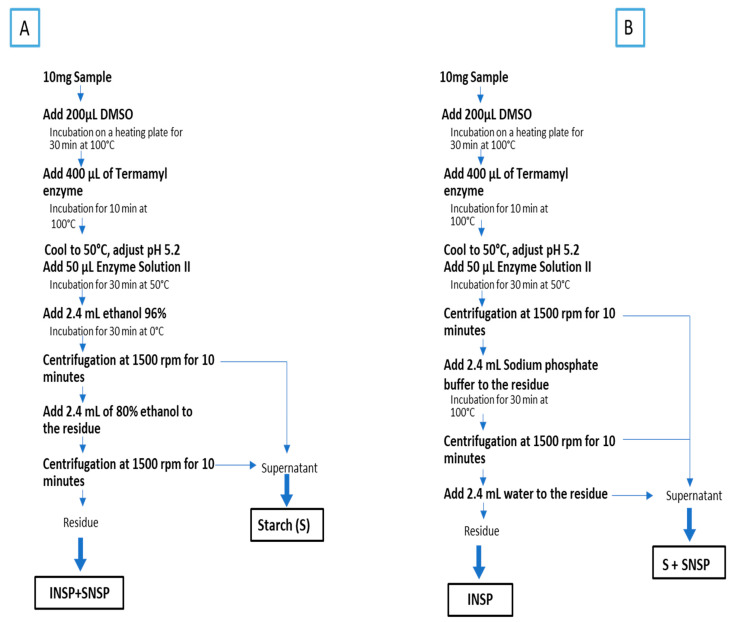
A schematic diagram of the modified Englyst method for the determination of starch and non-starch polysaccharide (NSP) fractions in *Satureja* species. Portion (**A**) allows simultaneous determination of total NSP (INSP + SNSP) and starch content, while Portion (**B**) specifically isolates insoluble NSPs (INSPs). Soluble NSPs (SNSPs) are calculated as the difference between total NSPs and insoluble NSPs. DMSO; dimethyl sulfoxide; S; starch; INSP; insoluble non-starch polysaccharide; SNSP; soluble non-starch polysaccharide.

Non-starch polysaccharides (NSPs) were determined using a modified Englyst method [22]. Two analytical portions (A and B) were prepared from each alcohol-insoluble residue (AIR) sample. Portion A was used to determine total NSP and starch content, while Portion B was used to quantify insoluble NSPs (INSPs). Soluble NSPs (SNSPs) were calculated as the difference between total NSPs and INSPs (Figure 1).

The modified Englyst-GC method was selected over alternative approaches such as AOAC enzymatic–gravimetric methods for two reasons: (1) it provides detailed monosaccharide profiles for each fiber fraction, enabling structural inferences essential for chemotype classification, whereas gravimetric methods yield only total fiber mass; and (2) it offers practical applicability for large-scale screening, as the analysis of 22 populations in triplicate required a robust method suitable for high-throughput comparative studies. However, a methodological consideration of the Englyst approach is that SNSP quantification by difference inherently propagates analytical uncertainty from both TNSP and INSP measurements, unlike gravimetric methods that directly quantify the soluble fraction.

**Enzymatic Hydrolysis.** For each portion, 10.0 mg (±0.1 mg) of AIR was accurately weighed and dispersed in 200 μL of analytical-grade dimethyl sulfoxide (DMSO). The suspension was incubated at 100 °C for 30 min in a heating block to ensure complete sample dispersion and initial starch gelatinization. Subsequently, 400 μL of heat-stable α-amylase (Termamyl 120 L, ≥120 KNU/g, Novo Nordisk, Bagsværd, Denmark) was added, and samples were incubated at 100 °C for an additional 10 min to facilitate starch gelatinization and initiate α-1,4-glycosidic bond hydrolysis. Following the initial α-amylase treatment, samples were cooled to 50 °C, and the pH was adjusted to 5.2 ± 0.1 using 0.5 M sodium acetate buffer. Fifty microliters of Enzyme Solution II (containing amyloglucosidase [EC 3.2.1.3] and bacterial protease in 0.5 M acetate buffer, pH 5.2) was added, and incubation continued at 50 °C for 30 min. This step ensured complete hydrolysis of starch to glucose by cleaving both α-1,4 and α-1,6 glycosidic bonds, while simultaneously digesting proteins that could interfere with subsequent NSP analysis.

**Total NSP and Starch Determination (Portion A).** Following enzymatic treatment, samples were cooled to room temperature, and 2.4 mL of ice-cold 96% (*v*/*v*) ethanol was added. The mixture was incubated at 0 °C for 30 min to precipitate NSP while maintaining glucose in solution. Samples were then centrifuged at 1500× *g* for 10 min at 4 °C, and the supernatant (containing liberated glucose) was carefully transferred to a clean tube and designated as Supernatant A1. The residue was resuspended in 2.4 mL of 80% (*v*/*v*) ethanol, thoroughly mixed, and centrifuged under the same conditions. The second supernatant (Supernatant A2) was combined with Supernatant A1 for starch quantification by the anthrone colorimetric method. The final residue, containing total NSPs (INSPs + SNSPs), was dried under nitrogen and prepared for monosaccharide composition analysis.

**Insoluble NSP Determination (Portion B).** Following identical enzymatic treatment as described above, samples were cooled to room temperature and centrifuged directly at 1500× *g* for 10 min at 4 °C. The supernatant was discarded. The residue was treated with 2.4 mL of sodium phosphate buffer (0.2 M, pH 7.0) and incubated at 100 °C for 30 min with periodic vortexing to extract soluble NSP. After centrifugation at 1500× *g* for 10 min, the supernatant (Supernatant B1) was collected. The residue was washed with 2.4 mL of deionized water, vortexed, and centrifuged again under the same conditions. The second supernatant (Supernatant B2) was combined with Supernatant B1; this combined supernatant contained both starch (as glucose) and soluble NSPs and was reserved for anthrone analysis. The final residue, representing insoluble NSPs (INSPs), was dried under nitrogen and prepared for monosaccharide analysis.

#### 2.3.2. Monosaccharide Composition Analysis

Neutral sugar (NS) composition was determined by gas–liquid chromatography following the two-step acid hydrolysis method of Saeman et al. [23]. After hydrolysis, monosaccharides were derivatized to alditol acetates and analyzed by gas chromatography [24]. A HP 6890 Plus gas chromatograph (Hewlett-Packard, Palo Alto, CA, USA) fitted with a 30 m × 250 μm × 0.20 μm capillary column (SP-2330, Supelco, Bellefonte, PA, USA) was used. The carrier gas was helium with a constant flow of 2.2 mL/min. Injection was performed in splitless mode. The oven temperature was held at 50 °C for 2 min after injection, then programmed to 180 °C at 35 °C/min, held at 180 °C for 5 min, then immediately increased to 220 °C at 5 °C/min, and held at 220 °C for 22 min. Total run was 40.7 min. The injector temperature was 250 °C, and the flame ionization detector (FID) was at 300 °C. Neutral sugars L-arabinose (Ara), D-xylose (Xyl), D-mannose (Man), D-galactose (Gal), and D-glucose (Glc) were identified. myo-Inositol was used as internal standard. Uronic acids (UA) were quantified by the m-hydroxybiphenyl method on both Portion A and Portion B residues [25,26]. Approximately 5 mg of each residue was hydrolyzed with concentrated sulfuric acid in an ice bath, and after colorimetric reaction with tetraborate/sulfuric acid reagent and m-hydroxybiphenyl reagent, absorbance was measured at 520 nm. UA content was expressed as galacturonic acid equivalents. The total NSPs was calculated as the sum of individual neutral sugars and uronic acids from Portion A residue. INSPs was calculated as the sum of neutral sugars and uronic acids from Portion B residue. SNSPs was calculated by difference (Total NSP − INSP). All determinations were performed in triplicate, and results were expressed as g/100 g dry matter.

### 2.4. Statistical Analysis

Statistical analyses were performed using STATGRAPHICS Centurion XVI for univariate comparisons and R version 4.3.0 with additional packages for multivariate analyses. For carbohydrate composition analysis, one-way analysis of variance (ANOVA) was used to evaluate differences among populations and species for total non-starch polysaccharides (TNSPs), insoluble non-starch polysaccharides (INSPs), soluble non-starch polysaccharides (SNSPs), and starch content. For monosaccharide composition analysis, independent sample comparisons were conducted between *S. nervosa* (n = 15) and *S. graeca* (n = 6) for each monosaccharide within each fiber fraction. *S. barceloi* was excluded from statistical comparisons due to insufficient sample size (n = 1) and reported descriptively only. The single *S. barceloi* population provides preliminary compositional data for this endemic species but does not permit assessment of intraspecific variation or robust interspecific statistical comparisons. Therefore, all statements regarding *S. barceloi* in the Results and Discussion sections are strictly descriptive and should not be interpreted as representing species-level characteristics. Welch’s *t*-tests (assuming unequal variances) were applied for two-group comparisons to account for unequal sample sizes and potential variance heterogeneity. Multiple testing correction was applied using the Bonferroni method to control family-wise error rate across the 18 monosaccharide-fraction combinations (6 monosaccharides × 3 fractions). The corrected significance level was set at α = 0.05/18 = 0.0028. Multivariate analysis: Principal component analysis (PCA) was performed using the prcomp() function to evaluate chemical differentiation patterns among populations using standardized data. Hierarchical cluster analysis was conducted using Ward’s linkage method with Euclidean distance (hclust() function) to identify chemical chemotypes. K-means clustering was applied using the kmeans() function to validate hierarchical clustering results, with optimal cluster number determined using the elbow method, silhouette analysis, and gap statistic from the factoextra package. All analyses were performed in triplicate, and results are expressed as mean ± standard deviation on a dry weight basis. For multivariate analyses, statistical significance was set at *p* < 0.05, while for multiple univariate comparisons, the Bonferroni-corrected significance level (*p* < 0.0028) was applied.

## 3. Results and Discussion

### 3.1. Dietary Fiber Composition of Species Populations

The comprehensive analysis of non-starch polysaccharide composition across 22 populations of three *Satureja* species revealed significant inter- and intraspecies variability in polysaccharide content (Table 1). Both *S. nervosa* (n = 15) and *S. graeca* (n = 6) demonstrated remarkably similar and elevated mean total non-starch polysaccharide (TNSP) content at 21.52 ± 3.02 g/100 g DW (CV = 14.0%) and 21.27 ± 3.72 g/100 g DW (CV = 17.5%), respectively. At the same time, the Tunisian endemic *S. barceloi* (n = 1) showed substantially lower levels at 13.76 ± 0.58 g/100 g DW. Direct comparison of these TNSP values with other Lamiaceae species or aromatic herbs from other families was not feasible, as the NSP method, which quantifies cellulose, hemicellulose, and pectin separately from starch and resistant starch, has not been systematically applied to culinary herbs in the published literature. Research on Lamiaceae predominantly focuses on phytochemical composition (essential oils, phenolic compounds, flavonoids) rather than cell wall polysaccharide characterization, resulting in an absence of comparable NSP data for most medicinal and culinary herbs. Notably, while Lamiaceae seeds such as chia (*Salvia hispanica*) and basil (*Ocimum basilicum*) have been extensively studied for their fiber content, reporting total dietary fiber values of 37.47 g/100 g DW and 40.85 g/100 g DW respectively [27], these values represent total dietary fiber (including resistant starch and lignin) rather than isolated NSP fractions measured by the Englyst method. Furthermore, these analyses focus on seeds rather than the aerial parts (leaves, stems, flowers) used as culinary herbs. This dual disparity, methodological (total dietary fiber vs. NSP) and botanical (seeds vs. aerial tissues), precludes meaningful direct comparisons and further limits the ability to contextualize the NSP profiles of *Satureja* aerial parts within the broader Lamiaceae family.

The fiber fractionation revealed remarkably balanced insoluble-to-soluble (I:S) polysaccharide ratios of 1.33 ± 0.56 for *S. nervosa*, 0.95 ± 0.40 for *S. graeca*, and 1.35 for *S. barceloi*. Most remarkably, several populations exhibited extraordinary soluble fiber proportions exceeding 50% of total fiber content: SN24 (58.0%), SN23 (57.7%), SN19 (55.5%), SN1 (50.5%), SN20 (49.1%), and SN17 (48.8%) from *S. nervosa*, plus SG21 (53.5%), SG18 (51.3%), and SG5 (51.1%) from *S. graeca*, with the exceptional outlier SG4 achieving an unprecedented 79.7% soluble fiber proportion (I:S ratio = 0.25). These values contrast markedly with reported fiber compositions in other plant materials. Green, leafy vegetables typically contain 11.3–36.0% soluble fiber (predominantly ~25%, I:S ~3:1) [28]. Common fruits and vegetables show limited soluble fiber proportions: apples (~30%), cooked carrots (~41%), and cooked broccoli (~40%), while certain fruits demonstrate higher proportions, including navel oranges (~58%), grapefruit (~65%), peaches (~46%), and prunes (~55%) [29]. The *Satureja* values also substantially exceed other Lamiaceae family members, including chia seeds (7–15%) and basil seeds (~21%) [30,31], approaching concentrations typically observed only in specialized mucilage-producing seed materials such as *psyllium* (*Plantago ovata*, 70–78% soluble fiber) [32,33].

However, while mucilage-containing seeds demonstrate concentrated soluble heteropolysaccharides (60–70% of mucilage content), their mucilage fraction represents only 6–12% of total seed weight [33]. In contrast, the high SNSP proportions in *Satureja* (mean values: *S. nervosa* 45.0 ± 8.9%; *S. graeca* 53.7 ± 12.6%) occur throughout the leaf material, constituting primary harvestable biomass, offering distinct practical advantages for large-scale fiber extraction, including greater biomass yield per cultivation area and multiple harvests per growing season [34,35].

Starch content analysis revealed consistent moderate levels across species, with mean values of 4.33 ± 2.01 g/100 g DW for *S. nervosa*, 4.44 ± 2.22 g/100 g DW for *S. graeca*, and 4.65 ± 0.30 g/100 g DW for *S. barceloi*, representing 28–33% lower values than *Thymus serpyllum* (6.5 g/100 g DW) [20]. The total carbohydrate profiles reached 25.85 ± 4.44 g/100 g DW for *S. nervosa*, 25.70 ± 5.32 g/100 g DW for *S. graeca*, and 18.41 g/100 g DW for *S. barceloi*, with fiber components overwhelmingly dominating the carbohydrate fraction (83.2%, 82.7%, and 74.7%, respectively). The coefficients of variation observed across fiber fractions reveal differential environmental and genetic control: starch exhibited the highest variability (CV = 46.4% in *S. nervosa*, 50.0% in *S. graeca*), followed by I:S ratio (CV = 42.5% and 41.4%, respectively), INSP (CV = 24.7% and 36.0%), and SNSP (CV = 20.5% and 10.6%), while total fiber content remained most stable (CV = 14.0% and 17.5%). Exceptional intraspecific variation was observed, with *S. nervosa* populations exhibiting TNSP ranges from 17.77 g/100 g DW (SN10) to 27.77 g/100 g DW (SN13), SNSP percentages from 25.9% (SN14) to 58.0% (SN24), and I:S ratios from 0.72 (SN24) to 2.86 (SN14), while *S. graeca* populations showed even more dramatic variation including the extreme soluble fiber specialist SG4 (79.7% SNSP, I:S = 0.25, TNSP = 13.56 g/100 g) versus more balanced types like SG3 (38.4% SNSP, I:S = 1.60, TNSP = 23.88 g/100 g. This intraspecific variation enables targeted selection for specific functional applications based on compositional similarity to characterized polysaccharides: high-soluble fiber populations (SG4, SN24, SN23, SG21) for applications similar to cholesterol-binding pectins [36,37], balanced profiles (SG3, SN7, SN13) for dietary supplementation, and high-total fiber types (SN13, SN7, SG3) for satiety-inducing bulking agents [9].

### 3.2. Monosaccharide Composition of Dietary Fiber Fractions in Tunisian Satureja Species

The monosaccharide analysis revealed striking compositional patterns that challenge conventional expectations for herbaceous plant cell walls (Table 2 and Table S2). Statistical comparison between *S. nervosa* and *S. graeca* using independent-samples Welch’s *t*-tests (α = 0.05) across all three fiber fractions detected no significant differences for any of the 20 testable monosaccharide comparisons, with the closest approach to significance being INSP arabinose (10.3 ± 2.4% vs. 12.0 ± 1.9%, *p* = 0.113, ns). This remarkable statistical similarity between geographically separated populations spanning Tunisia’s diverse bioclimatic zones suggests highly conserved cell wall biosynthetic pathways that maintain consistent polysaccharide architecture despite substantial environmental variation [38]. Most remarkably, all three *Satureja* species exhibited uronic acid dominance rather than the glucose dominance typical of most plant tissues, with uronic acids constituting the single most significant monosaccharide component in TNSP fractions: 42.9 ± 8.7% in *S. nervosa*, 48.3 ± 11.9% in *S. graeca*, and 52.5% in *S. barceloi*, substantially exceeding glucose levels (29.1 ± 5.5%, 26.4 ± 5.2%, and 35.2%, respectively) and creating Uro:Glc ratios (1.48, 1.83, 1.49) that dramatically exceed typical herbaceous plants, where glucose generally predominates [39,40]. This uronic acid enrichment intensifies in soluble fractions where SNSP uronic acids reach 74.6 ± 8.5% (*S. nervosa*), 75.3 ± 10.7% (*S. graeca*), and 80.1% (*S. barceloi*), creating extreme Uro:Glc ratios (11.17, 10.62, 4.90), indicating that *Satureja* soluble fiber consists overwhelmingly of acidic pectic polysaccharides. This compositional profile is similar to that of well-characterized pectic polysaccharides for which cholesterol binding, metal chelation, and pH-dependent gel formation have been documented [36,37], distinguishing these species from neutral polysaccharide-dominated fiber sources.

In contrast to the uronic acid-dominated total and soluble fractions, insoluble fiber fractions exhibited an expected glucose predominance characteristic of cellulose-rich cell walls, with INSP glucose constituting 47.2 ± 6.2% (*S. nervosa*), 48.4 ± 8.0% (*S. graeca*), and 49.2% (*S. barceloi*)—remarkably similar values, confirming conserved cellulose biosynthesis across species—accompanied by substantial xylose (17.0 ± 6.5%, 15.3 ± 3.8%, 6.9%) and arabinose (10.3 ± 2.4%, 12.0 ± 1.9%, 8.5%) representing hemicellulosic polysaccharides, while uronic acids remain present at moderate levels (16.3 ± 12.0%, 15.9 ± 12.7%, 31.8%), indicating pectin incorporation into insoluble cell wall matrices [38,39]. The arabinose–xylose ratios in the SNSP fractions (2.04 in *S. nervosa*, 1.22 in *S. graeca*, 2.36 in *S. barceloi*) suggest enhanced arabinoxylan branching compared to in the INSP fractions (0.60, 0.78, 1.23). Similar branching patterns in other plant arabinoxylans have been associated with enhanced water-holding capacity and favorable fermentation characteristics [41]. The (Ara + Gal)/Rha ratio provides critical insights into rhamnogalacturonan-I (RG-I) side-chain density, where high values indicate extensive arabinan and galactan substitution. This is exemplified by *S. nervosa* and *S. graeca*, which exhibited substantial ratios (7.91 and 8.89 in TNSP, respectively), signifying a highly branched pectic architecture with approximately 6–9 neutral sugar residues per rhamnose, a level of substitution that approaches highly substituted commercial pectins [42]. In stark contrast, *S. barceloi* exhibited a complete absence of rhamnose across all fractions, precluding ratio calculation and suggesting a fundamental divergence in pectin structure. This indicates that the pectin of *S. barceloi* is not based on a typical RG-I backbone but is likely dominated by homogalacturonan (HG), a structural paradigm supported by studies of other Lamiaceae species [43]. Consequently, the extreme uronic acid accumulation in *S. barceloi* (52.5% TNSP, 80.1% SNSP) most likely derives from unbranched homogalacturonan chains rather than the substituted RG-I structures of its congeners. However, this structural interpretation represents a compositional inference based on monosaccharide ratios rather than direct structural determination. Confirmation would require complementary analyses (NMR spectroscopy, methylation analysis, size-exclusion chromatography). This distinct HG-dominated architecture, a cornerstone of the primary plant cell wall [44], has direct implications for its functional properties, potentially leading to superior, calcium-mediated gel-forming ability and distinct metal-binding characteristics compared to the more branched pectins.

The coefficients of variation reveal extraordinary compositional plasticity, particularly in soluble fractions, with SNSP glucose exhibiting extreme variability (CV = 85.5% in *S. nervosa*, 82.3% in *S. graeca*) and SNSP xylose demonstrating the highest variability (CV = 88.9% and 100.9%), while SNSP arabinose showed substantial variation (CV = 62.9% and 75.9%), contrasting sharply with the moderate stability of TNSP glucose (CV = 19.1% and 19.6%) and the remarkable constancy of SNSP uronic acid (CV = 11.4% and 14.2%), indicating that while overall uronic acid biosynthesis remains constitutively regulated, creating the consistent high-uronic-acid phenotype, the neutral sugar composition responds dynamically to environmental or developmental factors [45]. Individual population analysis revealed remarkable compositional diversity within species (Table S2), with *S. nervosa* populations exhibiting dramatic SNSP variation including extreme-uronic acid specialists like SN19 (87.4% uronic acid, 3.2% glucose, 4.0% arabinose) and SN1 (83.7% uronic acid, 0.7% glucose, 7.0% arabinose) representing nearly pure pectic polysaccharides, contrasted with more balanced profiles like SN17 (70.9% uronic acid, 18.5% glucose, 5.0% arabinose) and glucose-enriched populations like SN16 (69.6% uronic acid, 14.4% glucose, 5.4% arabinose). Notably, SNSP arabinose content ranged from 3.6 to 4.0% (SN24, SN19) to 23.1% (SN13), with high-arabinose populations exhibiting compositional similarity to characterized prebiotic, water-soluble polysaccharides [46]. Population SN8 displayed exceptional SNSP galactose content (7.6%), approximately two-fold higher than typical populations [36]. Among *S. graeca* populations, SG5 exhibited elevated SNSP arabinose (11.2%), SG6 showed exceptional SNSP rhamnose (3.9%), and SG21 demonstrated the highest *S. graeca* SNSP arabinose (11.4%) and xylose (12.9%), with the extreme outlier SG4 showing minimal SNSP arabinose (1.3%) and xylose (1.1%) combined with exceptional TNSP uronic acid (69.8%), the highest among all populations. The single *S. barceloi* population (SB) exhibits extremely low SNSP neutral sugars (arabinose 0.8%, xylose 0.3%, galactose 0%, rhamnose 0%) combined with exceptional uronic acid (80.1%), representing the most compositionally extreme acidic SNSP profile documented in this study and warranting immediate conservation priority and expanded sampling to determine whether this profile is representative of the species [47,48].

The statistical equivalence between *S. nervosa* and *S. graeca* in monosaccharide composition (*p* > 0.05 for all comparisons) indicates conserved cell wall biosynthetic pathways, despite geographic separation [49]. The high population-level variation within *S. nervosa* and *S. graeca* represents metabolic diversity that may be relevant for breeding programs targeting specific fiber profiles [50,51]. The unique compositional profile of *S. barceloi* (80.1% uronic acid SNSP, 0% rhamnose) warrants further taxonomic and conservation investigation [52,53].

### 3.3. Inter- and Intraspecific Variability in Dietary Fiber Composition of Satureja Populations: A Principal Component Analysis Approach

**Figure 2:**
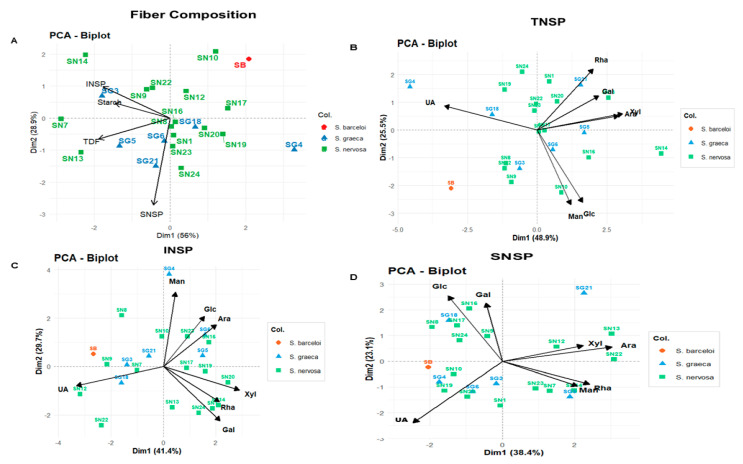
PCA biplots of dietary fiber composition in 22 *Satureja* populations. (**A**) Fiber composition (84.9% variance), (**B**) TNSP (74.4% variance), (**C**) INSP (71.1% variance), and (**D**) SNSP (61.5% variance explained). Green squares: *S. nervosa*, blue triangles: *S. graeca*, red circles: *S. barceloi* (Tunisian endemic). Black arrows indicate variable loadings. Populations cluster by fiber chemotype rather than species, demonstrating greater intraspecific than interspecific variation.

Principal component analysis across four complementary datasets progressively revealed the chemical architecture underlying fiber composition in 22 *Satureja* populations, with cumulative variance explanation increasing systematically from individual components to integrated profiles: SNSP explained 61.5% of variation (PC1: 38.4%, PC2: 23.1%), INSP captured 71.1% (PC1: 41.4%, PC2: 29.7%), and TNSP accounted for 74.4% (PC1: 48.9%, PC2: 25.5%), while the comprehensive Fiber Composition biplot explained 84.5% of total variance (PC1: 56.0%, PC2: 28.5%), demonstrating that multivariate fiber profiles differentiate populations more effectively than individual polysaccharide fractions. The integrated Fiber Composition biplot (Figure 2A) revealed taxonomic–compositional independence: TNSP, INSP, and Starch aligned closely, indicating strong positive correlations among structural fiber components, while SNSP oriented orthogonally, reflecting statistical independence from insoluble fiber accumulation. Populations distributed across the ordination space according to multivariate fiber profiles rather than taxonomic identity, with *S. nervosa* dispersing from insoluble-dominant profiles (SN7, SN13, SN14, SN9) through balanced soluble–insoluble partitioning (SN1, SN8, SN16, SN17, SN20, SN19, SN12) to soluble-enriched variants (SN24, SN23); *S. graeca* ranges from the extreme outlier SG4, positioned in the far-right, through balanced intermediate positions (SG18, SG6) to elevated soluble fractions (SG21, SG5), and the Tunisian endemic *S. barceloi* (SB) is positioned within the range of widespread *S. nervosa* populations rather than forming a distinct endemic cluster, demonstrating that intraspecific chemical variation dominates interspecific differentiation.

Total dietary fiber ordination (Figure 2B) revealed populations distributed along orthogonal chemical axes: the horizontal axis separated uronic acid-dominated populations (SG4, SB, SG18, SN8), through intermediate variants (SN7, SN17, SG5, SG6, SN16), to neutral sugar-enriched populations (SN24, SN1, SN19, SN22, SG21, SN12, SN14, SN20), while the vertical axis captured rhamnose–galactose enrichment versus mannose–glucose dominance. The soluble non-starch polysaccharide biplot (Figure 2C) revealed four distinct chemical clusters: glucose–galactose-enriched (SN8, SN16, SN17, SN24, SG18), arabinose–xylose-enriched (SN12, SN13, SN22, SG21), uronic acid specialists (SB, SG4, SN10, SN19), and moderate monosaccharide levels (SN1, SN7, SN23, SN9, SG5, SG6). The insoluble polysaccharide ordination (Figure 2D) segregated populations into an extreme mannose accumulator (SG4), glucose–arabinose-enriched cluster (SN8, SN9, SN17, SG3, SG5), xylose–rhamnose–galactose-dominated group (SN14, SN19, SN20, SN13), and uronic acid specialists (SB, SN22). Critically, both *S. nervosa* and *S. graeca* populations dispersed across all chemical groups in each ordination, while *S. barceloi* clustered with other uronic acid specialists rather than forming species-specific clusters, confirming that intraspecific diversity in polysaccharide architecture substantially exceeded interspecific differentiation. The orthogonal positioning of SNSP and INSP vectors in PCA space (Figure 1A) indicates these fiber fractions vary independently across samples, reflecting distinct biosynthetic regulation: soluble pectic polysaccharides depend primarily on UDP-sugar precursor pools and pectin methylesterase activity, while insoluble cellulosic matrices are synthesized by cellulose synthase complexes [54,55].

### 3.4. Hierarchical Clustering Reveals Cross-Species Chemotypes

While PCA revealed the primary axes of compositional variation and orthogonal relationships between fiber fractions, hierarchical clustering provides a quantitative assessment of population relationships through Euclidean distance metrics, enabling the determination of the magnitude of chemical differentiation between compositional groups.

**Figure 3:**
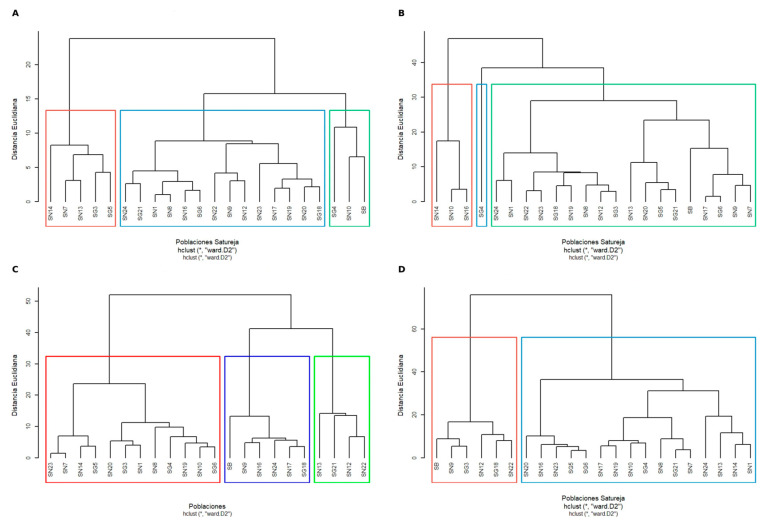
Hierarchical clustering dendrograms of dietary fiber composition in 22 *Satureja* populations using Ward’s D2 method and Euclidean distance. (**A**) Comprehensive fiber composition, including total non-starch polysaccharides (TNSPs), insoluble non-starch polysaccharides (INSPs), soluble non-starch polysaccharides (SNSPs), and starch content, revealing three major clusters (red, blue, green boxes) separating at approximately 15 units of Euclidean distance. (**B**) total non-starch polysaccharide (TNSP) monosaccharide composition showing three clusters separating at 30–35 units, with populations grouping by monosaccharide profiles rather than taxonomic identity. (**C**) Soluble non-starch polysaccharides (SNSPs), displaying three clusters based on pectic polysaccharide architecture, with populations distributed by soluble fiber chemotype independently of species boundaries. (**D**) Insoluble non-starch polysaccharides (INSPs) exhibiting the highest Euclidean distances (approximately 60 units) among all datasets, reflecting maximum chemical differentiation in cell wall polysaccharide composition. Colored boxes indicate major cluster groupings identified at biologically meaningful dissimilarity thresholds. All dendrograms demonstrate taxonomic intermixing where *Satureja nervosa*, *S. graeca*, and the Tunisian endemic *S. barceloi* (SB) cluster by fiber chemotype rather than phylogenetic relationships, corroborating PCA ordination patterns (Figure 2) and confirming that intraspecific variation exceeds interspecific differentiation across all fiber compositional levels. Ward’s D2 agglomeration method minimizes within-cluster variance while maximizing between-cluster separation, providing robust discrete classifications complementary to the continuous variance gradients visualized through principal component analysis.

Hierarchical clustering dendrograms (Figure 3A–D), using Ward’s D2 method and Euclidean distance, corroborated and extended the multivariate patterns revealed by PCA ordinations, demonstrating that *Satureja* populations cluster by fiber chemotype rather than taxonomic identity, with cluster membership varying substantially between datasets, indicating that different fiber fractions capture independent dimensions of biochemical diversity. The comprehensive fiber composition dendrogram (Figure 3A) revealed three major clusters, separating at approximately 15 units of Euclidean distance: a left cluster (SN14, SN7, SN13, SG3, SG5), a central cluster (SN24, SG21, SN1, SN8, SN16, SG6, SN22, SN9, SN12, SN23, SN17, SN19, SN20, SG18), and a right cluster (SG4, SN10, SB). *S. nervosa* populations were distributed across all three clusters, *S. graeca* was similarly dispersed, and the Tunisian endemic *S. barceloi* (SB) clustered with *S. nervosa* population SN10 and *S. graeca* population SG4. The TNSP dendrogram (Figure 3B) revealed fundamentally different clustering relationships, with three clusters separating at approximately 33 units: a far-left cluster (SN14, SN10, SN16), an isolated outlier (SG4), and a large right cluster containing the majority of populations, including SB integrated with *S. nervosa* and *S. graeca* populations. The SNSP dendrogram (Figure 3C) demonstrated yet another clustering pattern, with three groups separating at approximately 32 units: a left cluster (SN23, SN7, SN14, SG5, SN20, SG3, SN1, SN8, SG4, SN19, SN10, SG6), a central cluster (SB, SN9, SN16, SN24, SN17, SG18), and a right cluster (SN13, SG21, SN12, SN22), with cluster membership shifting substantially compared to both Figure 3A,B. The INSP dendrogram (Figure 3D) revealed the most divergent clustering structure, with two major groups separating at approximately 55 units of Euclidean distance, the highest separation distance among all four datasets: a left cluster (SB, SN9, SG3, SN12, SG18, SN22) and a large right cluster (SN20, SN16, SN23, SG5, SG6, SN17, SN19, SN10, SG4, SN8, SG21, SN7, SN24, SN13, SN14, SN1).

Comparing across all four dendrograms reveals dynamic cluster membership where individual populations shift positions depending on which fiber fraction is examined: SN14 traverses from left cluster (Figure 3A) to far-left cluster (Figure 3B) to left cluster (Figure 3C) to right cluster (Figure 3D), spanning the entire hierarchical space; SN10 shifts from right cluster (Figure 3A) to far-left cluster (Figure 3B) to left cluster (Figure 3C) to right cluster (Figure 3D), demonstrating positional plasticity; SG4 occupies outlier or peripheral positions across analyses as an isolated outlier (Figure 3B), reflecting its extreme biochemical profile; and most critically, the Tunisian endemic *S. barceloi* (SB) demonstrates remarkable positional plasticity by shifting from right cluster (Figure 3A) to large right cluster (Figure 3B) to central cluster (Figure 3C) to left cluster (Figure 3D), traversing the entire hierarchical space and clustering with different *S. nervosa* and *S. graeca* populations depending on the analyzed fraction. The Euclidean distance analysis revealed striking differences between fiber fractions. INSP showed the largest distances (55 units), while SNSP and TNSP exhibited considerably smaller values (15–33 units). This pattern reflects fundamental differences in polysaccharide organization. In plant cell walls, insoluble structural polysaccharides form discrete architectural types. Type I walls (dicots) feature pectin-rich, xyloglucan-crosslinked networks, whereas Type II walls (grasses) employ pectin-poor, arabinoxylan-based structures [56]. Because these architectural categories are biochemically distinct, INSP composition shows discontinuous variation, manifested as large Euclidean distances, rather than gradual transitions. Soluble fractions, by contrast, lack these structural constraints. Soluble pectins exist as free polymers that can vary continuously in monosaccharide composition without architectural thresholds [57]. This explains why SNSPs and TNSPs exhibit smaller Euclidean distances, representing gradual compositional gradients rather than discrete structural types.

### 3.5. K-Means Clustering Validates Chemotype Classification Through Spatial Partitioning

**Figure 4:**
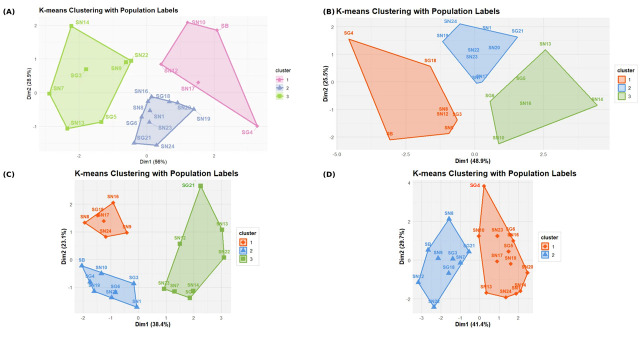
K-means clustering validates chemotype classification in *Satureja* populations. K-means analysis overlaid on PCA ordinations for (**A**) comprehensive fiber composition (k = 3, 84.9% variance: pink, blue, green clusters), (**B**) total non-starch polysaccharides (k = 3, 74.4% variance: orange, blue, green clusters), (**C**) soluble non-starch polysaccharides (k = 3, 61.5% variance: orange, blue, green clusters), and (**D**) insoluble non-starch polysaccharides (k = 2, 71.1% variance: orange and blue clusters only). The shift from three-cluster solutions (**A**–**C**) to a two-cluster solution (**D**) reflects fundamental cell wall architectural constraints (Type I versus Type II dichotomy).

To validate and refine chemotype classifications, we performed K-means clustering analysis overlaid on PCA ordinations, partitioning populations into mutually exclusive clusters by minimizing within-cluster variance. The comprehensive fiber composition K-means analysis (Figure 4A) identified three optimal clusters explaining 84.9% of total variance (Dim1: 56.0%, Dim2: 28.9%). The pink cluster contained five populations (SN10, SN12, SN17, SB, SG4), the blue cluster contained ten populations (SN16, SG18, SN8, SN1, SG6, SN23, SN20, SN19, SG21, SN24), and the green cluster contained seven populations (SN14, SG3, SN7, SN9, SN22, SN13, SG5). TNSP K-means analysis (Figure 4B) revealed three spatial clusters explaining 74.4% of variance (Dim1: 48.9%, Dim2: 25.5%). The orange cluster contained seven populations (SG4, SG18, SN8, SN12, SG3, SN9, SB), the blue cluster contained eight populations (SN24, SN1, SN19, SN22, SN20, SN23, SN17, SG21), and the green cluster contained seven populations (SN13, SG5, SG6, SN16, SN14, SN10). SNSP K-means analysis (Figure 4C) identified three clusters explaining 61.5% of variance (Dim1: 38.4%, Dim2: 23.1%). The orange cluster contained six populations (SN16, SG18, SN8, SN17, SN24, SN9), the blue cluster contained eight populations (SB, SN10, SG4, SN19, SG6, SN20, SG3, SN1), and the green cluster contained eight populations (SG21, SN13, SN12, SN22, SN23, SN7, SN14, SG5). The INSP K-means analysis (Figure 4D) revealed a fundamental departure from other datasets: only two optimal clusters explained 71.1% of variance (Dim1: 41.4%, Dim2: 29.7%), in contrast to the three clusters observed in comprehensive, TNSP, and SNSP analyses. The orange cluster contained thirteen populations (SG4, SN10, SN23, SG6, SN16, SG5, SN17, SN19, SN20, SN13, SN24, SN14, SN1), and the blue cluster contained nine populations (SN8, SB, SN9, SG3, SN7, SG21, SG18, SN12, SN22).

The consistent identification of only two optimal clusters for INSP, across both hierarchical (Figure 3D) and K-means (Figure 4D) methods, contrasts sharply with three clusters observed for all other fiber fractions. This pattern suggests a fundamental constraint in plant cell wall architecture where insoluble polysaccharide composition exhibits bistable states corresponding to alternative structural solutions [38,56].

This architectural constraint explains why INSP consistently shows only two clusters (k = 2). The dichotomy reflects the fundamental Type I versus Type II cell wall organization, well-documented in plant biology [38,39]. Type I walls (dicot pattern) utilize pectin-rich, xyloglucan-crosslinked networks optimized for extensibility during symplastic growth, whereas Type II walls (grass pattern) employ arabinoxylan-crosslinked structures optimized for load bearing in apoplastic expansion [56]. Intermediate architecture would be mechanically compromised, lacking both the flexibility necessary for cell elongation and the rigidity required for structural integrity [58]. This mechanical incompatibility constrains populations to occupy one of two discrete compositional states, with natural selection eliminating intermediate phenotypes. Soluble polysaccharides behave differently. Because they are not part of the structural cell wall matrix, they can vary continuously in composition without affecting mechanical properties [57]. This explains why soluble fractions (TNSPs, SNSPs) show three clusters with gradual transitions between them, while insoluble polysaccharides (INSPs) show only two distinct types with a clear gap between them.

The concordance between K-means spatial partitioning, hierarchical dendrogram topology, and PCA ordination patterns provides robust statistical validation of observed chemotype groupings. These three mathematically independent multivariate methods operate through fundamentally different algorithms: variance maximization in PCA, agglomerative similarity in hierarchical clustering, and iterative centroid optimization in K-means. Their convergence on similar population groupings demonstrates that observed patterns represent genuine biochemical discontinuities rather than analytical artifacts [59]. Multivariate analyses demonstrated that populations group primarily by polysaccharide composition rather than taxonomic identity. Uronic acid-dominated populations (SG4, SB, SG18, SN8) exhibit compositional similarity to commercial pectins characterized by cholesterol-binding capacity, metal chelation, and pH-dependent gel formation [36,37], representing potential sources for industrial pectin extraction. High neutral-sugar populations (SN24, SN1, SN22, SG21) show compositional similarity to fermentable fiber residues supporting colonic microbiota [60,61]. Arabinose–xylose-enriched populations (SN13, SN12, SN22, SG21) exhibit compositional similarity to prebiotic arabinoxylans that selectively stimulate beneficial gut microbiota [41,46]. Populations with balanced intermediate monosaccharide profiles (SN7, SN9, SG3, SG5, SN16) provide general dietary fiber profiles suitable for broad nutritional supplementation [9].

All three *Satureja* species were distributed across all chemotypes, with individual populations shifting cluster assignments depending on which fiber fraction was analyzed. The Tunisian endemic *S. barceloi* clustered with different *S. nervosa* and *S. graeca* populations when classified by comprehensive fiber content, total polysaccharide composition, soluble fraction, or insoluble fraction, demonstrating multidimensional chemical variation that cannot be captured by single compositional variables. *S. nervosa* populations were distributed across all chemotype groups in every analysis, *S. graeca* populations were similarly dispersed, and *S. barceloi* clustered with uronic acid specialists rather than forming a species-specific group. This pattern demonstrates that intraspecific polysaccharide diversity substantially exceeds interspecific differentiation [62], consistent with adaptive differentiation models where polysaccharide composition is more responsive to localized selective pressures (edaphic, climatic, or biotic factors) than to phylogenetic constraints, providing metabolic flexibility in fiber biosynthetic pathways [57].

The Tunisian endemic *S. barceloi* clusters with widespread congeners rather than exhibiting unique fiber composition, yet its extreme uronic acid content (52.5% TNSP, 80.1% SNSP) combined with the complete absence of rhamnose suggests a fundamentally different pectin structure, likely dominated by homogalacturonan rather than the rhamnogalacturonan-I architecture observed in *S. nervosa* and *S. graeca* [44]. This biochemically distinctive profile warrants further taxonomic and conservation investigation [45,46]. More broadly, germplasm conservation and breeding strategies may benefit from prioritizing populations with rare metabolic phenotypes alongside traditional taxonomic criteria [61]. Population SG4 exhibits an extreme outlier profile across multiple analyses (79.7% SNSP proportion, I:S ratio = 0.25), representing greater compositional diversity than many other populations, while geographically dispersed arabinose–xylose specialists (SN13, SG21, SN22, SN12) maintain polysaccharide architectures potentially valuable for prebiotic applications. This metabolic diversity-based approach to conservation may better preserve functional biochemical variation than taxonomic status alone [63], particularly where intraspecific compositional variation substantially exceeds interspecific differences [62].

## 4. Conclusions

This comprehensive analysis of 22 populations across three Tunisian *Satureja* species reveals significant chemical diversity transcending taxonomic boundaries. Multivariate analyses identified three distinct chemotypes in soluble and total fiber fractions, with insoluble fiber exhibiting a fundamental two-cluster dichotomy reflecting Type I versus Type II cell wall architecture. This demonstrates that chemical composition predicts functional properties more reliably than morphological classification.

Exceptional populations warrant immediate attention for functional food development. *S. nervosa* populations SN24 (58.0% SNSP), SN23 (57.7% SNSP), and SN19 (55.5% SNSP), along with *S. graeca* populations SG4 (79.7% SNSP), SG21 (53.5% SNSP), and SG18 (51.3% SNSP), exhibited outstanding soluble fiber proportions exceeding 50%, comparable to established high-fiber sources. Additionally, populations SN13 and SN7 demonstrated exceptional total dietary fiber content (27.8 and 26.8 g/100 g DW, respectively), while populations SN13, SN12, SN22, and SG21 exhibited specialized arabinose–xylose enrichment, valuable for prebiotic applications. These populations represent valuable genetic resources for developing nutraceutical ingredients targeting cholesterol reduction, glycemic control, and prebiotic effects.

Endemic *S. barceloi* exhibited extreme uronic acid dominance (52.5% TNSP, 80.1% SNSP) with complete absence of rhamnose, representing a unique homogalacturonan-dominated pectin architecture distinct from the rhamnogalacturonan-I structures in *S. nervosa* and *S. graeca*. The reported *S. barceloi* fiber composition represents a single population (n = 1) and may not reflect species-level characteristics. Confirmation of this unique biochemical phenotype requires sampling of multiple geographically distinct populations to distinguish species-specific traits from population-level variation.

High intraspecific variation (CV: 14.0–50.0%) exceeding interspecific differences indicates substantial potential for population-level breeding programs. The consistent identification of two clusters in insoluble fiber across multiple analytical methods reveals that cell wall architecture constrains populations to Type I or Type II configurations, requiring pathway-specific breeding approaches: cellulose synthase modification for xylose-rich populations versus pectin biosynthesis gene targeting for uronic acid-rich populations.

Future research should focus on validating chemotype functional significance through bioactivity assays (cholesterol-binding capacity, glycemic response modulation, prebiotic fermentation profiles), confirming pectin structural inferences through direct analytical methods (NMR spectroscopy, methylation analysis, size-exclusion chromatography) to definitively distinguish homogalacturonan from rhamnogalacturonan-I architectures, investigating the genetic basis of fiber composition variation through transcriptomic or genomic approaches, and establishing field trials to assess environmental stability of chemotype classifications across multiple growing seasons. The validated chemotype-based classification system provides a robust framework for systematic evaluation of *Satureja* species as functional dietary fiber sources and establishes methodological precedents for population-level phytochemical screening in other medicinal plant families.

## Figures and Tables

**Table 1:** Dietary fiber composition of *Satureja* species populations collected from different bioclimatic zones in Tunisia.

			Content (g/100 g DW)				
Population	TNSP	INSP	SNSP	Starch	Total CHO	I:S Ratio	SNSP%
***S. nervosa***							
SN1	21.37 ± 0.23	10.59 ± 0.10	10.78 ± 0.33	3.98 ± 0.15	25.35	0.98	50.5
SN7	27.28 ± 0.16	16.46 ± 0.04	10.82 ± 0.20	7.89 ± 0.36	35.17	1.52	39.7
SN8	21.59 ± 0.07	11.34 ± 0.24	10.24 ± 0.31	3.67 ± 0.29	25.25	1.11	47.4
SN9	21.53 ± 0.74	13.17 ± 0.17	8.35 ± 0.91	5.67 ± 0.08	27.20	1.58	38.8
SN10	17.77 ± 0.11	12.41 ± 0.09	5.36 ± 0.02	2.12 ± 0.36	19.89	2.32	30.2
SN12	19.64 ± 0.97	11.57 ± 0.84	8.07 ± 0.13	3.94 ± 0.06	23.58	1.43	41.1
SN13	27.77 ± 0.03	15.29 ± 0.32	12.48 ± 0.35	5.64 ± 0.23	33.41	1.23	44.9
SN14	25.62 ± 0.21	18.99 ± 0.03	6.63 ± 0.24	5.69 ± 0.31	31.31	2.86	25.9
SN16	21.98 ± 0.48	12.14 ± 0.38	9.84 ± 0.10	2.43 ± 0.01	24.41	1.23	44.8
SN17	17.83 ± 0.05	9.13 ± 0.11	8.70 ± 0.06	2.19 ± 0.03	20.02	1.05	48.8
SN19	18.55 ± 0.09	8.26 ± 0.24	10.29 ± 0.15	2.43 ± 0.13	20.97	0.80	55.5
SN20	20.30 ± 0.21	10.34 ± 0.12	9.96 ± 0.09	1.69 ± 0.38	22.00	1.04	49.1
SN22	19.56 ± 0.37	11.11 ± 0.32	8.46 ± 0.05	8.02 ± 0.46	27.58	1.31	43.2
SN23	20.22 ± 0.19	8.56 ± 0.00	11.66 ± 0.19	6.20 ± 0.34	26.42	0.73	57.7
SN24	21.81 ± 0.15	9.16 ± 0.54	12.65 ± 0.38	3.32 ± 0.28	25.13	0.72	58.0
**Mean ± SD**	**21.52 ± 3.02**	**11.90 ± 2.94**	**9.62 ± 1.98**	**4.33 ± 2.01**	**25.85 ± 4.44**	**1.33 ± 0.56**	**45.0 ± 8.9**
**CV (%)**	**14.0**	**24.7**	**20.5**	**46.4**	**17.2**	**42.5**	**19.7**
***S. graeca***							
SG3	23.88 ± 0.46	14.70 ± 0.89	9.18 ± 0.43	7.59 ± 0.49	31.47	1.60	38.4
SG4	13.56 ± 0.07	2.75 ± 0.04	10.81 ± 0.10	2.08 ± 0.10	15.64	0.25	79.7
SG5	23.60 ± 0.70	11.54 ± 0.14	12.06 ± 0.56	7.38 ± 0.63	30.98	0.96	51.1
SG6	23.12 ± 0.07	12.07 ± 0.02	11.05 ± 0.09	2.52 ± 0.08	25.65	1.09	47.8
SG18	19.75 ± 0.07	9.62 ± 0.28	10.14 ± 0.35	3.66 ± 0.19	23.41	0.95	51.3
SG21	23.68 ± 1.00	11.00 ± 0.32	12.68 ± 0.68	3.40 ± 0.10	27.08	0.87	53.5
**Mean ± SD**	**21.27 ± 3.72**	**10.28 ± 3.70**	**10.99 ± 1.16**	**4.44 ± 2.22**	**25.70 ± 5.32**	**0.95 ± 0.40**	**53.7 ± 12.6**
**CV (%)**	**17.5**	**36.0**	**10.6**	**50.0**	**20.7**	**41.4**	**23.6**
***S. barceloi***							
SB	13.76 ± 0.58	7.90 ± 0.62	5.86 ± 0.04	4.65 ± 0.30	18.41	1.35	42.6

Values represent mean ± standard deviation (g/100 g dry weight) based on triplicate analyses (n = 3). TNSP = Total non-starch polysaccharide; INSP = insoluble non-starch polysaccharide; SNSP = soluble non-starch polysaccharide; Total CHO = Total carbohydrates (TNSP + Starch); I:S Ratio = insoluble–soluble fiber ratio; SNSP% = soluble fiber as percentage of total dietary fiber; DW = dry weight: CV = Coefficients of variation. Values are mean ± standard deviation (n = 3).

**Table 2:** Comprehensive monosaccharide composition and structural analysis of dietary fiber fractions in three *Satureja* species collected from different bioclimatic zones across Tunisia.

Species	n	Fraction	Structural Polysaccharides			Pectic Polysaccharides					Key Structural Ratios			Coefficient of Variation (%)			
			Glc	Xyl	Man	Ara	Rha	Gal	Fuc	Uro	Uro:Glc	Ara:Xyl	(Ara + Gal)/Rha	Glc	Ara	Xyl	Uro
***S. nervosa***	**15**	**TNSP**	29.1 ± 5.5 (22–39) ^a^	11.0 ± 4.0 (6–22) ^a^	3.3 ± 0.3 (3–4) ^a^	9.1 ± 1.6 (8–14) ^a^	1.5 ± 1.0 (0–2) ^a^	3.1 ± 0.9 (2–4) ^a^	0.0 ± 0.0 ^a^	42.9 ± 8.7 (22–54) ^a^	1.48	0.82	7.91	19.1	18.0	35.8	20.3
		**INSP**	47.2 ± 6.2 (35–58) ^a^	17.0 ± 6.5 (6–29) ^a^	4.2 ± 1.1 (3–7) ^a^	10.3 ± 2.4 (6–14) ^a^	1.5 ± 1.4 (0–4) ^a^	3.6 ± 1.9 (0–8) ^a^	0.0 ± 0.0 ^a^	16.3 ± 12.0 (3–39) ^a^	0.35	0.60	9.46	13.2	23.8	38.5	73.6
		**SNSP**	6.7 ± 5.7 (1–18) ^a^	3.9 ± 3.5 (0–11) ^a^	2.3 ± 1.9 (0–8) ^a^	8.1 ± 5.1 (4–23) ^a^	1.8 ± 1.6 (0–5) ^a^	2.6 ± 1.9 (0–8) ^a^	0.0 ± 0.0 ^a^	74.6 ± 8.5 (58–87) ^a^	11.17	2.04	6.05	85.5	62.9	88.9	11.4
***S. graeca***	**6**	**TNSP**	26.4 ± 5.2 (17–33) ^a^	9.4 ± 3.7 (4–14) ^a^	3.2 ± 0.5 (3–4) ^a^	8.3 ± 2.5 (4–11) ^a^	1.3 ± 1.1 (0–3) ^a^	3.0 ± 1.1 (1–4) ^a^	0.0 ± 0.0 ^a^	48.3 ± 11.9 (39–70) ^a^	1.83	0.88	8.89	19.6	30.4	39.7	24.6
		**INSP**	48.4 ± 8.0 (37–58) ^a^	15.3 ± 3.8 (11–21) ^a^	5.3 ± 1.9 (4–9) ^a^	12.0 ± 1.9 (10–15) ^a^	0.0 ± 0.0 ^a^	3.1 ± 1.7 (0–5) ^a^	0.0 ± 0.0 ^a^	15.9 ± 12.7 (2–33) ^a^	0.33	0.78	-	16.4	15.5	24.9	79.7
		**SNSP**	7.1 ± 5.8 (0–16) ^a^	4.8 ± 4.8 (0–13) ^a^	1.8 ± 0.9 (1–3) ^a^	5.9 ± 4.4 (1–11) ^a^	2.4 ± 2.1 (0–5) ^a^	2.7 ± 1.6 (1–5) ^a^	0.0 ± 0.0 ^a^	75.3 ± 10.7 (56–85) ^a^	10.62	1.22	3.62	82.3	75.9	100.9	14.2
***S. barceloi***	**1**	**TNSP**	35.2 ± 0.0 (35–35) ^d^	4.1 ± 0.0 (4–4) ^d^	3.1 ± 0.0 (3–3) ^d^	5.2 ± 0.0 (5–5) ^d^	0.0 ± 0.0 ^d^	0.0 ± 0.0 ^d^	0.0 ± 0.0 ^d^	52.5 ± 0.0 (52–52) ^d^	1.49	1.27	-	0.0	0.0	0.0	0.0
		**INSP**	49.2 ± 0.0 (49–49) ^d^	6.9 ± 0.0 (7–7) ^d^	3.7 ± 0.0 (4–4) ^d^	8.5 ± 0.0 (8–8) ^d^	0.0 ± 0.0 ^d^	0.0 ± 0.0 ^d^	0.0 ± 0.0 ^d^	31.8 ± 0.0 (32–32) ^d^	0.65	1.23	-	0.0	0.0	0.0	0.0
		**SNSP**	16.4 ± 0.0 (16–16) ^d^	0.3 ± 0.0 (0–0) ^d^	2.3 ± 0.0 (2–2) ^d^	0.8 ± 0.0 (1–1) ^d^	0.0 ± 0.0 ^d^	0.0 ± 0.0 ^d^	0.0 ± 0.0 ^d^	80.1 ± 0.0 (80–80) ^d^	4.90	2.36	-	0.0	0.0	0.0	0.0

Data represent mean ± standard deviation (minimum-maximum range) of monosaccharide percentages determined by gas–liquid chromatography following acid hydrolysis, reduction, and acetylation. Each value represents the mean of two technical replicates per population. Sample sizes: *S. nervosa* (n = 15), *S. graeca* (n = 6), *S. barceloi* (n = 1). Structural ratios include: arabinose–xylose (Ara:Xyl); glucose–non-glucose, calculated as glucose % ÷ (arabinose + xylose + mannose + rhamnose + galactose + fucose)% (Glc:Non-Glc), and rhamnose–galactose (Rha:Gal). Coefficients of variation (CV) indicate population-level compositional variability. Statistical methodology: Species compositional differences were evaluated separately within each fiber fraction (TNSP, INSP, SNSP) using independent-samples *t*-tests between *S. nervosa* and *S. graeca* (df = 19, α = 0.05). This within-fraction approach correctly identifies species-specific differences for each fiber type rather than comparing across different polysaccharide fractions. *S. barceloi* (n = 1) was excluded from statistical testing and is reported descriptively only. Different superscript letters (a, d) within the same fiber fraction and monosaccharide column indicate significant differences between species (*p* < 0.05). Five significant differences were identified: TNSP mannose, INSP mannose, INSP arabinose, INSP rhamnose, and SNSP arabinose, with three remaining significant after Bonferroni correction (*p* < 0.003). Abbreviations: TNSP = total non-starch polysaccharides; INSP = insoluble non-starch polysaccharides; SNSP = soluble non-starch polysaccharides. Fiber profile characteristics describe predicted functional properties based on monosaccharide composition and structural ratios, with implications for bulking capacity, water-binding properties, and prebiotic potential.

## Data Availability

The original contributions presented in this study are included in the article/Supplementary Material. Further inquiries can be directed to the corresponding authors.

## Supplementary Materials

The following supporting information can be downloaded at: https://www.mdpi.com/article/10.3390/foods15030525/s1, Table S1: Location and Main Ecological Traits of 22 Tunisian populations of *Satureja nervosa*, *S. barceloi*, and *S. graeca*; Table S2: Monosaccharide composition of dietary fiber fractions in *Satureja* populations.
