# Supplementary material for: Inter- and Intraspecific Variability in Non-Starch Polysaccharide Composition of Satureja Species from Tunisia: Implications for Functional Food Development
Source: Foods. 2026 Feb 3;15(3):525. doi: 10.3390/foods15030525 (PMC12897150; doi:10.3390/foods15030525)
Supplement: Supplementary file 1 [file foods-15-00525-s001.zip › foods-4103702-supplementary.pdf]

Inter- and Intraspecific Variability in Non-Starch Polysaccharide Composition of *Satureja* Species from Tunisia: Implications for Functional Food Development

Anhar Raadani [1,†], Amel Hamdi [2,*,†], Islem Yangui [3,4], Ana Jiménez-Araujo [2], Rocío Rodríguez-Arcos [2],
Imen Ben Elhadj Ali [1,5], Rafael Guillén-Bejarano [2,*] and Chokri Messaoud [1]

[1] Laboratory of Nanobiotechnology and Valorisation of Medicinal Phytoresources, National Institute of Applied Sciences and Technology, Department of Biology, Carthage University, B.P. 676, Tunis Cedex 1080, Tunisia; raadanianharbio@gmail.com (A.R.); imenbenelhadjali@yahoo.fr (I.B.E.A.); chok.messaoud@yahoo.fr (C.M.)
[2] Phytochemicals and Food Quality Group, Instituto de la Grasa, Consejo Superior de Investigaciones Científicas (CSIC), Pablo de Olavide Universitary Campus, Building 46, Carretera de Utrera Km 1, 41013 Seville, Spain; araujo@ig.csic.es (A.J.-A.); rrodri@ig.csic.es (R.R.-A.)
[3] Department of Plant Protection and Biological Sciences, University of Sousse, Higher Institute of Agronomy of Chott-Meriem, Sousse 4042, Tunisia; yanguiislam@gmail.com
[4] Laboratory of Management and Valorization of Forest Resources, University of Carthage, National Research Institute for Rural Engineering, Water and Forests, Ariana 2080, Tunisia
[5] Higher Institute of Biotechnology of Beja, Jendouba University, Beja 9000, Tunisia
* Correspondence: ahamdi@ig.csic.es (A.H.); rguillen@ig.csic.es (R.G.-B.)
† These authors contributed equally to this work.

Table S1. Location and Main Ecological Traits of 22 Tunisian populations of *Satureja nervosa*, *S. barceloi*, and *S. graeca*.

Bioclimatic zone [a]	Population code	Geographic region	Site	Q_2 [c] coefficient	Latitude	Longitude	Altitude (m)	Rainfall (mm/year)
Lh	SN23	Nord West	El feija	96.5	36°48’N	8°18’E	800	800-1200
Lh	SN13	Nord West	Sajnane	93.4	36°40’N	10°23’E	450	800-1200
Sh	SN7	High Tell	El Hairech Jeb. Mt. [b]	64.52	36°10’N	8°4’E	400	500 - 600
Sh	SN8	Cap Bon	Korbous	65.64	36°50’ N	10°35’ E	400	500 - 600
Sh	SG4	High Tell	Beja	$63.5 < Q_2 < 96.5$	36°43’30" N	9°10’55" E	252	500 - 600
Sh	SN14	Cap Bon	Abderrahmen Jeb. Mt.	65.64	36°40’ N	10°41’E	420	500 - 600
Sh	SN10	Cap Bon	Zaouiet El Megaîz	86.69	36°50’ N	10°50’ E	60	500 - 600
Sh	SN9	Cap Bon	Takelsa	81.96	36°47’ N	10°38’ E	123	500 - 600
Sh	SB	Nord East	Ghar El Melh	$63.5 < Q_2 < 96.5$	37°19'55"N	9°51'01"E	215	500 - 600
Sh	SN24	Nord West	Joumine	$63.5 < Q_2 < 96.5$	36°55'32"N	9°23'20"E	305	500 - 600
Sh	SN19 SG21	Nord West	Ain Jamel	$63.5 < Q_2 < 96.5$	36°27'26"N	9°14'54"E	400	500 - 600
Usa	SG3	High Tell	Medjez-el-Bab	$44.5<Q_2<63.5$	36°38'58"N	9°36'44"E	51	400 - 500
Usa	SN1	Nord West	Sidi Thabet	$44.5<Q_2<63.5$	36°54'31"N	10°2'33"E	14	400 - 500
Usa	SG5	North Central	El Amayem	45.72	36°21’ N	9°54’ E	600	400 - 500
Usa	SN12	Tunisian Dorsale	Nadhour	$44.5<Q_2<63.5$	36°0719"N	10°05'34"E	170	400 - 500
Usa	SN16	Tunisian Dorsale	Jdidi Jeb. Met.*	$44.5<Q_2<63.5$	36°23'8"N	10°7'23"E	300	400 - 500

Usa	SN22	Tunisian Dorsale	Hammam Zriba	44.5 < Q_2 < 63.5	36°23'8" N	10°7'23" E	300	400 - 500
Usa	SN17	High Tell	El Krib	51.4	36°19'38" N	9°8'7" E	481	400 - 500
Usa	SG18	High Tell	Nebeur	44.5<Q_2<63.5	36°17'56" N	8°45'24" E	426	400 - 500
Msa	SG6	Tunisian Dorsale	Serj Jeb. Met.*	35.5<Q_2<44.5	35°40'N	9°35'E	610	400 - 500
Msa	SG20	High Tell	Essers	43.24	36°76'N	9°40'E	610	400 - 500

[a] Bioclimatic zone: Lh: lower-humid.Sh: sub-humid; Usa: upper semi-arid; Msa: Mean semi-arid;
[b] Jeb. Mt.: Jebel Mountain.
[c] Q_2: Emberger's pluviothermic coefficient (1966). $Q_2 = 2000P/ M^2 - m^2$ where *P* is the mean of annual rainfall (mm), *M* (K°) is the mean of maximal temperatures for the warmest month (July) and *m* is the mean of minimal temperatures for the coldest month (February). *P*, *M* and m values for each site were calculated for the period from 1953 to 2007 (Data provides by the Tunisian National Institute of Meteorology.

Table S2. Monosaccharide composition of dietary fiber fractions in *Satureja* populations

	% composition								
**Population**	**Fraction**	**Arabinose**	**Rhamnose**	**Fucose**	**Xylose**	**Mannose**	**Galactose**	**Glucose**	**Uronic acid**
Satureja nervosa									
**SN1**	TDF	8.8±0.2	2.2±0.1	0.0±0.0	14.6±0.1	3.0±0.0	3.4±0.3	22.0±0.1	46.0±0.3
	INSP	10.5±0.1	2.2±0.0	0.0±0.0	26.6±0.2	3.6±0.1	5.7±0.0	43.7±0.9	7.7±1.3
	SNSP	7.0±0.4	2.3±0.1	0.0±0.0	2.8±0.0	2.4±0.1	1.1±0.6	0.7±0.2	83.7±0.4
**SN7**	TDF	7.6±0.1	1.9±0.0	0.0±0.0	12.9±0.1	3.2±0.0	1.9±0.2	29.7±0.2	42.7±0.0
	INSP	8.1±0.0	0.0±0.0	0.0±0.0	16.5±0.0	3.9±0.0	2.2±0.0	48.4±0.1	20.8±0.1
	SNSP	6.9±0.3	4.9±0.0	0.0±0.0	7.5±0.3	2.0±0.0	1.6±0.6	1.1±0.1	76.0±0.5
**SN8**	TDF	7.7±0.4	0.0±0.0	0.0±0.0	5.8±0.0	3.6±0.0	3.6±0.3	28.7±0.2	50.7±0.2
	INSP	10.4±0.3	0.0±0.0	0.0±0.0	10.2±0.3	6.7±0.1	0.0±0.0	48.8±1.2	23.9±1.8
	SNSP	4.7±0.7	0.0±0.0	0.0±0.0	0.9±0.1	0.2±0.0	7.6±0.8	6.4±0.6	80.3±0.8
**SN9**	TDF	8.3±0.2	0.0±0.0	0.0±0.0	7.6±0.3	3.6±0.0	1.9±0.0	31.9±0.8	46.6±1.3
	INSP	9.2±0.3	0.0±0.0	0.0±0.0	9.2±0.3	4.5±0.3	2.0±0.1	43.5±0.1	31.5±0.4
	SNSP	6.9±0.1	0.0±0.0	0.0±0.0	5.2±0.8	2.0±0.6	1.7±0.3	13.6±0.1	70.5±0.1
**SN10**	TDF	9.8±0.1	0.0±0.0	0.0±0.0	11.5±0.1	3.5±0.0	2.4±0.0	37.3±0.2	35.4±0.4
	INSP	12.3±0.1	0.0±0.0	0.0±0.0	14.2±0.1	4.9±0.0	2.3±0.0	52.0±0.4	14.4±0.7
	SNSP	4.2±0.0	0.0±0.0	0.0±0.0	5.1±0.0	0.5±0.0	2.7±0.0	3.3±0.0	84.2±0.0
**SN12**	TDF	8.3±0.1	0.0±0.0	0.0±0.0	7.3±0.0	3.7±0.2	2.4±0.1	27.9±1.4	50.4±1.8
	INSP	6.3±0.2	0.0±0.0	0.0±0.0	6.2±0.4	3.1±0.4	2.5±0.3	42.9±2.7	38.9±3.9
	SNSP	11.2±0.1	0.0±0.0	0.0±0.0	8.9±0.7	4.5±0.1	2.3±0.1	6.5±0.8	66.7±0.3
**SN13**	TDF	13.9±0.1	2.5±1.0	0.0±0.0	14.2±0.6	2.9±0.2	3.2±0.0	29.7±2.2	33.7±0.3
	INSP	6.4±0.2	1.6±0.0	0.0±0.0	23.8±0.0	2.9±0.0	3.6±0.3	48.3±1.1	13.4±1.5
	SNSP	23.1±1.0	3.6±2.3	0.0±0.0	2.4±0.7	2.9±0.5	2.6±0.3	6.8±4.8	58.5±0.0

**SN14**	TDF	10.7±0.1	2.4±0.0	0.0±0.0	21.7±0.1	3.8±0.0	4.3±0.5	35.6±0.3	21.6±0.1
	INSP	10.8±0.0	1.9±0.1	0.0±0.0	29.0±0.3	2.5±0.1	4.5±0.1	47.6±0.1	3.7±0.1
	SNSP	10.3±0.4	3.5±0.1	0.0±0.0	0.7±0.1	7.6±0.1	3.7±2.5	1.2±0.2	73.0±2.7
**SN16**	TDF	9.0±0.4	2.1±0.1	0.0±0.0	9.7±0.2	3.5±0.3	4.2±0.2	38.7±1.1	32.9±2.3
	INSP	11.8±0.3	2.9±0.0	0.0±0.0	16.0±0.5	5.0±0.1	2.8±0.2	58.3±2.6	3.1±3.8
	SNSP	5.4±0.6	1.2±0.2	0.0±0.0	2.0±0.1	1.5±0.5	5.8±0.0	14.4±0.2	69.6±1.2
**SN17**	TDF	7.6±0.2	2.1±0.0	0.0±0.0	9.4±0.0	3.0±0.0	3.0±0.1	34.1±0.1	40.7±0.2
	INSP	10.2±0.5	2.5±0.2	0.0±0.0	17.5±0.2	5.2±0.0	3.7±0.2	49.0±0.7	11.9±0.1
	SNSP	5.0±0.1	1.6±0.2	0.0±0.0	1.0±0.0	0.7±0.1	2.3±0.4	18.5±0.6	70.9±0.3
**SN19**	TDF	8.5±0.1	2.0±0.0	0.0±0.0	7.0±0.2	2.9±0.1	2.9±0.0	23.1±0.2	53.6±0.0
	INSP	14.1±0.4	4.3±0.1	0.0±0.0	14.3±0.1	4.4±0.1	3.6±0.1	47.8±1.4	11.5±2.0
	SNSP	4.0±0.3	0.2±0.1	0.0±0.0	1.1±0.1	1.7±0.2	2.4±0.1	3.2±0.0	87.4±0.1
**SN20**	TDF	10.0±0.1	2.1±0.0	0.0±0.0	9.6±0.1	3.0±0.1	4.0±0.0	27.8±0.2	43.4±0.0
	INSP	11.9±0.1	2.7±0.0	0.0±0.0	18.8±0.1	5.1±0.1	7.5±0.1	50.3±0.6	3.7±0.9
	SNSP	8.1±0.0	1.4±0.0	0.0±0.0	0.2±0.0	0.9±0.1	0.4±0.0	4.4±0.9	84.7±0.8
**SN22**	TDF	9.8±0.3	2.1±0.1	0.0±0.0	11.1±0.5	3.3±0.1	2.5±0.0	22.9±0.4	48.4±1.4
	INSP	6.8±0.4	1.2±0.1	0.0±0.0	10.8±0.3	2.7±0.1	4.0±0.2	36.5±0.9	38.0±1.8
	SNSP	13.8±0.0	3.1±0.1	0.0±0.0	11.5±0.8	4.0±0.2	0.5±0.1	4.9±0.2	62.0±1.3
**SN23**	TDF	8.9±0.1	2.0±0.0	0.0±0.0	12.8±0.0	3.2±0.1	2.0±0.3	24.1±0.1	47.0±0.2
	INSP	11.7±0.7	0.0±0.0	0.0±0.0	19.6±0.7	4.9±0.0	2.6±0.1	55.3±0.2	5.9±1.3
	SNSP	6.8±0.6	3.5±0.0	0.0±0.0	7.7±0.4	2.0±0.2	1.5±0.4	1.2±0.3	77.2±0.1
**SN24**	TDF	7.6±0.1	1.7±0.1	0.0±0.0	10.6±0.2	2.6±0.1	4.4±0.3	22.6±0.1	50.4±0.3
	INSP	13.3±1.2	2.5±0.1	0.0±0.0	22.1±1.1	4.1±0.2	6.5±0.4	34.8±2.0	16.8±5.1
	SNSP	3.6±0.4	1.1±0.1	0.0±0.0	2.2±0.3	1.6±0.1	3.0±0.6	13.9±0.6	74.6±1.0
Satureja graeca									
**SG3**	TDF	8.4±0.1	0.0±0.0	0.0±0.0	10.2±0.1	3.7±0.1	2.5±0.1	27.5±0.1	47.7±0.4
	INSP	10.3±0.7	0.0±0.0	0.0±0.0	11.6±0.6	4.6±0.0	2.9±0.1	43.8±2.2	26.9±3.5

	SNSP	5.4±0.6	0.0±0.0	0.0±0.0	8.1±0.3	2.4±0.4	1.8±0.1	1.4±0.5	81.0±0.9
**SG4**	TDF	4.1±0.0	1.3±0.1	0.0±0.0	3.9±0.0	2.7±0.0	1.2±0.1	17.0±0.1	69.8±0.3
	INSP	15.1±0.4	0.0±0.0	0.0±0.0	15.2±0.5	9.0±0.4	0.0±0.0	48.9±0.2	11.8±0.2
	SNSP	1.3±0.1	1.6±0.1	0.0±0.0	1.1±0.2	1.1±0.1	1.5±0.1	8.9±0.0	84.5±0.1
**SG5**	TDF	11.0±0.5	2.5±0.0	0.0±0.0	12.4±0.1	3.7±0.0	3.0±0.2	27.9±0.7	39.4±0.9
	INSP	10.8±0.1	0.0±0.0	0.0±0.0	20.8±0.2	4.4±0.1	4.8±0.1	56.6±0.4	2.6±0.4
	SNSP	11.2±0.9	4.9±0.1	0.0±0.0	4.4±0.7	3.2±0.1	1.3±0.4	0.5±0.1	74.5±0.1
**SG6**	TDF	7.6±0.0	1.9±0.0	0.0±0.0	9.8±0.0	3.5±0.3	3.4±0.2	32.8±0.5	40.9±0.7
	INSP	12.6±0.3	0.0±0.0	0.0±0.0	18.3±0.4	5.1±0.2	4.4±0.4	57.7±0.2	1.9±0.0
	SNSP	2.2±0.3	3.9±0.1	0.0±0.0	0.5±0.4	1.9±0.9	2.4±0.8	5.7±1.0	83.6±0.9
**SG18**	TDF	7.9±0.0	0.0±0.0	0.0±0.0	6.3±0.0	2.8±0.1	3.9±0.0	26.1±0.2	53.1±0.3
	INSP	12.5±0.3	0.0±0.0	0.0±0.0	10.9±0.5	3.7±0.1	3.6±0.3	36.7±0.8	32.6±2.1
	SNSP	3.6±0.0	0.0±0.0	0.0±0.0	1.9±0.2	1.8±0.3	4.1±0.3	16.1±0.5	72.5±1.3
**SG21**	TDF	10.9±0.7	2.0±0.0	0.0±0.0	13.9±0.3	2.8±0.1	4.2±0.2	27.0±1.1	39.1±2.4
	INSP	10.4±0.6	0.0±0.0	0.0±0.0	15.1±0.9	5.1±0.0	3.1±0.3	46.6±1.0	19.7±2.3
	SNSP	11.4±0.8	3.8±0.0	0.0±0.0	12.9±0.3	0.7±0.2	5.2±0.6	10.1±0.8	55.9±2.1
Satureja barceloi									
**SB**	TDF	5.2±0.0	0.0±0.0	0.0±0.0	4.1±0.1	3.1±0.2	0.0±0.0	35.2±1.7	52.5±2.1
	INSP	8.5±0.7	0.0±0.0	0.0±0.0	6.9±0.5	3.7±0.3	0.0±0.0	49.2±3.9	31.8±5.4
	SNSP	0.8±0.5	0.0±0.0	0.0±0.0	0.3±0.2	2.3±0.3	0.0±0.0	16.4±0.5	80.1±0.1

Monosaccharide composition (percentage of total sugars ± standard deviation) of total dietary fiber (TDF), insoluble non-starch polysaccharides (INSP), and soluble non-starch polysaccharides (SNSP) fractions in 22 populations of three *Satureja* species collected from different bioclimatic zones in Tunisia. Values represent mean percentages based on gas-liquid chromatography analysis following hydrolysis, reduction, and acetylation procedures according to Englyst et al. (1994).
